# Engagement With Conversational Agent–Enabled Interventions in Cardiometabolic Disease Self-Management: Systematic Review

**DOI:** 10.2196/67913

**Published:** 2025-09-18

**Authors:** Nick Kashyap, Ann Tresa Sebastian, Chris Lynch, Paul Jansons, Ralph Maddison, Tilman Dingler, Brian Oldenburg

**Affiliations:** 1 School of Psychology and Public Health La Trobe University Melbourne Australia; 2 Baker Department of Cardiovascular Research, Translation and Implementation La Trobe University Melbourne Australia; 3 Non-communicable Disease and Implementation Science laboratory Baker Heart and Diabetes Institute Melbourne Australia; 4 Institute for Physical Activity and Nutrition, School of Exercise and Nutrition Sciences Deakin University Melbourne Australia; 5 Department of Medicine, School of Clinical Sciences at Monash Health Monash University Melbourne Australia; 6 Delft University of Technology Delft The Netherlands

**Keywords:** user experience, cardiovascular disease, diabetes, chronic disease, chatbot, digital assistant, qualitative, digital health

## Abstract

**Background:**

Well-designed conversational agents can improve health care capacity to meet the dynamic and complex needs of people self-managing cardiometabolic diseases (CMD). However, a lack of empirical evidence on conversational agent–enabled intervention design features and their impact on engagement make it challenging to comprehensively evaluate effectiveness. This review synthesizes evidence on conversational agent–enabled intervention design features and how they impact on engagement to inform the development of more engaging conversational agent–enabled interventions that effectively help people with CMD to self-manage their condition.

**Objective:**

The aim of the study is to synthesize evidence pertaining to conversational agent–enabled intervention design features and their impact on engagement of people self-managing CMD.

**Methods:**

Searches were conducted in Ovid (MEDLINE), Web of Science, and Scopus databases. Inclusion criteria were primary research studies reporting on conversational agent–enabled interventions that included measures of engagement and included adults with CMD. Data extraction captured perspectives of people with CMD on various design features of conversational agent–enabled interventions.

**Results:**

Of 1366 studies identified for screening, 20 were included in the review. In total, 18 of these were qualitative or quasi-experimental evaluations of conversational agent–enabled intervention prototypes. Five domains of design features that impact user engagement with conversational agent–enabled interventions emerged: communication style, functionality, accessibility, visual appearance, and personality.

**Conclusions:**

Across all 5 domains, integrating redundancy and anthropomorphism were identified as effective strategies for improving engagement by increasing user autonomy and investment. Future research should adopt design strategies that are inclusive and adaptive to the diverse needs of users and aligned with the unique considerations relevant to conversational agent–enabled interventions.

**Trial Registration:**

PROSPERO CRD42023431579; https://tinyurl.com/3srmzw8f

**International Registered Report Identifier (IRRID):**

RR2-10.2196/52973

## Introduction

Supporting effective self-management is crucial for reducing both the health and economic impacts of cardiometabolic diseases (CMD) but is challenged by the dynamic and complex nature of CMDs and limitations faced by health care providers [1-3]. Encompassing a set of interrelated conditions that affect the cardiovascular system and metabolic health, such as hypertension and diabetes, CMDs are a significant health concern [4]. Globally, CMDs cause approximately 20 million deaths each year, with morbidity and mortality rates continuing to increase [5-7]. It has been estimated that nearly 80% of CMD deaths and a significant proportion of associated health care costs could be prevented with optimal self-management [2,8]. Self-management describes the ability of patients to monitor their symptoms and circumstances and understand how these factors affect health [9]. Due to the individualized and chronic course of CMDs, sustaining effective self-management requires people to continuously adjust to changing symptoms and personal circumstances [10-12]. For example, people may forget to take medications or understand changes in their medication schedule. Interventions supporting people self-managing CMDs must be continuously customized to align with the dynamic and complex experience of each individual [13]. However, limitations in time, resources, expertise, or confidence often hinder the capacity of health care providers to deliver this level of care [3,12].

Conversational agents, technologies that interact with people using natural language, can overcome some of the limitations faced by health care providers [14-16]. Conversational agents are typically accessed through app-based platforms, websites, or SMS text messaging services and can interact with the user via text, speech, or visually as embodied avatars. When incorporated into digital care delivery, conversational agents can enable continuous monitoring and alignment of interventions with the dynamic circumstances of people self-managing CMD through providing contextually relevant assistance [15,17]. Conversational agents can assess user comprehension, offer real-time emotional and motivational support, translate specialized language into familiar terms, integrate relevant dialogues with on-screen instructions, and provide voice-based interaction options [14,16]. For example, a person with hypertension receiving a reading of 150/95 mm Hg may not understand whether this requires immediate action, medication, or lifestyle changes. A multimodal conversational agent can assess their comprehension through dialogue (“What does this reading mean to you?”), provide personalized explanations based on their medication regimen, and offer contextual guidance while displaying visual aids showing normal versus elevated ranges. This is just one example of how a specific design feature, multimodality, can improve a person’s engagement with an intervention supporting them to self-manage their condition. Capabilities of agents have been advancing, as these technologies have undergone rapid progress since their emergence in the 1960s [18,19]. Despite this progress, the ongoing challenge of sustaining user engagement, a common issue in digital health, continues to limit the use of conversational agents in clinical contexts, since sustained interaction is key to their effectiveness [20-22].

The effectiveness of conversational agent–enabled interventions in supporting self-management is dependent on user engagement, which describes the depth and frequency of a user’s interactions with an intervention [23-25]. While engagement is a well-recognized concept, its application in digital health often varies based on different perspectives and methodologies for measurement [26-28]. For example, some researchers equate engagement directly with user activity, while others consider how well an intervention aligns with a user’s cognitive beliefs [27]. To achieve a change in health-related behaviors, digital health interventions require sufficient participant engagement so users not only understand and apply intervention content but also develop the skills, motivation, and confidence necessary to make sustainable lifestyle changes [24,29,30]. However, the low rates of engagement with most digital health interventions for self-management of chronic diseases are well known [21,31,32].

The design features of digital health interventions are important determinants of user engagement and are typically informed by theory and empirical evidence, such as the self-reference encoding model [25,33,34]. For example, researchers observed that when self-relevant cues were used explicitly to refer to users’ smoking habits, rather than using them implicitly to tailor content, improved smoking cessation outcomes occurred [23]. This work demonstrates both the benefits and the limitations of applying theory to design. In particular, reflecting on how the theory was applied in practice provided a more nuanced understanding of the role of self-relevant cues as a design feature.

The design of conversational agent–enabled interventions can benefit from empirical evidence on design features and their impacts on engagement in people self-managing CMDs [23,25]. Empirical evidence of this kind is sparse, primarily found in the evaluation of prototypes within qualitative and quasi-experimental studies [35-38]. This review fills this gap through a thematic synthesis that explicitly pairs individual design features with the engagement outcomes they influence. Synthesizing the empirical evidence could enable the recognition of broad trends, reduce bias in design reflections, and contribute to more nuanced applications of theories and models. This synthesis can facilitate the development of more engaging and effective conversational agent–enabled interventions for supporting people with CMDs to self-manage their condition.

## Methods

### Protocol and Registration

The methodology used in this study has been detailed in the protocol paper [39]. This review was conducted in accordance with the Cochrane Handbook for Systematic Reviews of Interventions and reported in accordance with the PRISMA (Preferred Reporting Items for Systematic Reviews and Meta-Analyses) checklist [40,41] (Multimedia Appendix 1). The review was initiated in May 2023 and registered in PROSPERO (CRD42023431579).

### Information Sources

We systematically searched Ovid (MEDLINE), Web of Science, and Scopus databases for relevant papers from inception until April 2024. Ovid (MEDLINE) was selected for its medical and health science focus to capture studies that included CMDs. Web of Science and Scopus were selected for their broader focus, which is useful for capturing research on conversational agents and engagement topics. We excluded certain databases after preliminary assessments. For example, Embase was excluded due to its significant content overlap with MEDLINE, and the Cochrane Library was excluded because it lacked a significant number of relevant exemplar papers found in the other databases. As this is a rapidly developing field, final searches were conducted prior to the submission of this manuscript. Reference list searches were conducted on all papers included in the full-text review.

Although IEEE Xplore and the ACM Digital Library do contain work on conversational agents, a scoping search showed that almost all health-related records from those sources were already captured in Ovid (MEDLINE), Web of Science, or Scopus. The unique papers we found in the computing databases focused on technical architecture and system development, with minimal reporting on end-user engagement, the central outcome of this review. Because our objective was to synthesize evidence on how broad design features influence participant engagement (regardless of the underlying algorithm), and not to catalogue implementation details, the search strategy was confined to the 3 multidisciplinary databases, Ovid (MEDLINE), Web of Science, and Scopus. This focused approach ensured capture of health-focused literature while maintaining feasibility, as our pilot searches confirmed that the selected databases provided comprehensive coverage of conversational agent research with engagement outcomes in health care populations.

### Search Strategy

An extensive set of search terms was used, which related to the 3 central topics of the review: CMD, conversational agents, and engagement. Boolean operators were used to combine search terms, including: (“Cardiovascular Diseases” [MeSH Terms] OR metabolic) AND (“conversational agent*” OR chatbot*) AND (accept* OR perceived). This approach was intended to yield a comprehensive collection of literature that explores the intersection of these central topics. The complete search strategy can be found in Multimedia Appendix 2.

### Eligibility Criteria

#### Population

Studies included adult participants (≥18 years of age) with a CMD diagnosis. Populations reported with comorbidities, such as mental health disorders or multiple CMDs, were also included.

#### Intervention

Studies reported on conversational agent–enabled interventions, which supported people with CMD to self-manage their condition. For example, by offering emotional or educational support or assisting individuals in monitoring their symptoms.

#### Outcomes

Studies reported on engagement outcomes, such as ratings, interviews, analytics, and focus groups.

#### Exclusion Criteria

Excluded from this study were technical feasibility studies without user engagement data, studies where CMD populations were not reported separately, and nonpeer-reviewed studies (theses, preprints, abstract only, etc).

### Study Design

Studies were primary research, including qualitative studies, quasi-experimental studies, and randomized controlled trials (RCTs). Reviews, editorials, protocols, and non-English publications were excluded.

### Data Management

All search results were imported into PaperPile (Paperpile LLC), and duplicates were automatically removed. The search results were then imported into Covidence (Veritas Health Innovation), where 53 more duplicates were removed. Covidence was used to store PDF files of papers considered during the full-text review and to conduct study selection, data extraction, and quality appraisal, with data then being exported to Microsoft Excel.

### Study Selection

During initial screening, 2 reviewers (NK and ATS) independently examined the titles and abstracts of all studies collected from the search strategy. Screening was based on the defined inclusion and exclusion criteria. Conflict was resolved by meetings between the 2 reviewers (NK and ATS). If a conflict was not able to be resolved, it was to be arbitrated by a third reviewer to achieve consensus, though this did not occur. Remaining studies were independently assessed by 2 reviewers (NK and ATS) in full-text screening, using the defined inclusion and exclusion criteria. Any conflict between 2 reviewers (NK and ATS) was settled during meetings, with arbitration by a third reviewer if required though this did not occur.

### Data Extraction

Data extraction from all included studies, including any supplementary material, was conducted and documented by 2 reviewers (NK and ATS) independently. Where further information was required, study authors were contacted for clarification. The data extraction form was designed to collect comprehensive information on how people with CMD engage with different design features in conversational agent–enabled interventions. Details included bibliographic information, study design, participants and population, intervention features, and how design features influenced engagement outcomes.

### Quality Appraisal

Due to the mixed study designs of the included literature, the following Joanna Briggs Institute critical appraisal tools were used: “Checklist for Qualitative Research,” “Checklist for Quasi-Experimental Studies,” and “Assessment of Risk of Bias for Randomized Controlled Trials” [42-44].

### Data Synthesis

An adapted version of the thematic synthesis analysis method developed by Thomas and Harden [45] was used. This method focused on data extracted from studies that detailed design features and engagement outcomes. During the initial phase, data were categorized under multiple domains. Following the extraction, domains were iteratively consolidated until further merging would compromise the descriptive accuracy of each dataset. Then, within each domain, data were categorized under multiple subdomains. Analysis of these domains and subdomains contributed to informing design choices for conversational agent–enabled interventions and identifying research gaps in how engagement is reported in the literature.

## Results

### Overview

**Figure 1:**
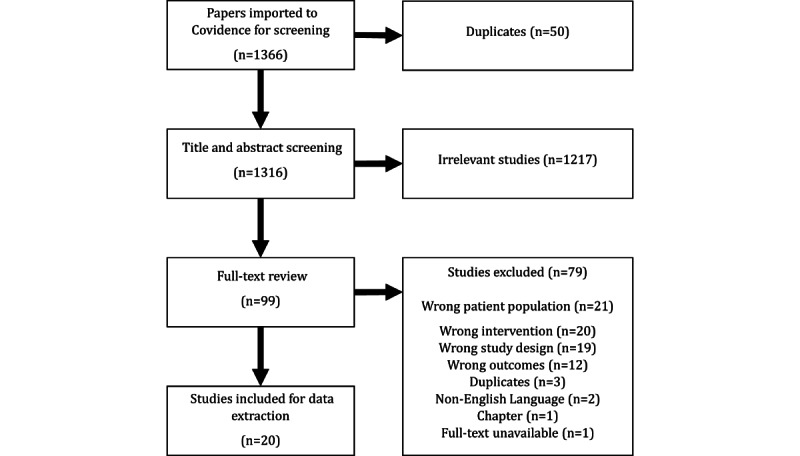
Flowchart illustrating the study selection process.

Of 1366 studies imported for screening, 20 studies were included in the review (Figure 1). All included studies were published between 2015 and 2023 (Table 1). In total, 10 studies focused on cardiovascular disease (heart failure, atrial fibrillation, acute coronary syndrome, hypertension, and stroke), 9 studies focused on diabetes (type 2 diabetes and gestational diabetes mellitus), and 1 study focused on chronic kidney disease (Table S1 in Multimedia Appendix 2) [35-38,46-62]. The sample sizes ranged from 8 to 187, and the age of the participants ranged from 21 to 87 years (Table S1 in Multimedia Appendix 2). A total of 13 of the conversational agents were embodied (ranging from simple, cartoon-like avatars in a digital interface to sophisticated, humanoid robots capable of mimicking human gestures and expressions), and 7 were chatbots (text-only interface; Table 1). Overall, studies using embodied agents revealed broader effects of design features on engagement outcomes than studies using chatbots. For further information about study designs, populations, and interventions, refer to Multimedia Appendix 2.

**Table 1:** Characteristics of included studies.

Author (year)	Study design	Conversational agent role	Conversational agent type, platform, and dialogue system	Measures of engagement (total sample size)
Apergi et al (2021) [37]	Quasi-experimental	To ask the patients the same series of questions related to their heart failure treatment and symptoms and provide feedback.	Embodied, app-based+Alexa, tree-based	Surveys, questionnaires, analytics (n=30)
Balsa et al (2019) [46]	Qualitative	To assist older people with type 2 diabetes mellitus in medication adherence and lifestyle changes.	Embodied, app-based, tree-based	Self-administered questionnaire (n=28)
Balsa et al (2020) [47]	Qualitative	To support older people with type 2 diabetes mellitus in medication adherence and lifestyle changes.	Embodied, app-based, tree-based	Questionnaire, open questions, diaries, digital notes, telephone follow-ups (n=20)
Baptista et al (2020) [36]	Quasi-experimental	To deliver self-management education and support to adults with type 2 diabetes mellitus.	Embodied, app-based, tree-based	Web-based surveys, interview (n=93)
Cheng et al (2018) [35]	Qualitative	To allow for a less cumbersome way for patients with geriatric type 2 diabetes mellitus to effectively adhere to guidelines.	Embodied, web-based+Google Home, AI^a^-based	Subjective tests for qualitative assessment of effectiveness and satisfaction, gathering feedback from experts and potential users (n=10)
Echeazarra et al (2021) [48]	Randomized controlled trial	To help patients with hypertension to self-monitor their blood pressure.	Chatbot, messaging service, tree-based	Satisfaction survey (n=112)
Epalte et al (2023) [38]	Qualitative	To counsel, educate, and train people who had a stroke and their family on stroke, rehabilitation, care, and other related issues.	Chatbot, app-based, AI-based	Semistructured interviews (n=12)
Gingele et al (2023) [49]	Qualitative	To evaluate patient’s health status, provide patient education, and improve their ability to communicate with heart failure nurses.	Embodied, app-based, AI-based	System usability scale, self-developed patient satisfaction scale, general feedback form (n=37)
Gong et al (2020) [50]	Randomized controlled trial	To provide more accessible and engaging self-management support, monitoring, and coaching to adults with type 2 diabetes mellitus in Australia.	Embodied, app-based, tree-based	Data analytics, process evaluation (n=187)
Guhl et al (2020) [51]	Quasi-experimental	To augment patient-centered health care by providing health education, monitoring, and problem-solving for users.	Embodied, app-based, tree-based	Measures, self-reports (n=120)
Kimani et al (2016) [52]	Quasi-experimental	To provide education on atrial fibrillation and promote adherence to daily heart rhythm monitor readings.	Embodied, app-based, tree-based	Data analytics, self-report scale, semistructured interview (n=16)
Magnani et al (2017) [53]	Quasi-experimental	To promote education, motivation, and monitor patient’s symptoms and adherence to behaviors.	Embodied, app-based, tree-based	Qualitative interviews, questionnaire (n=31)
Nassar et al (2025) [54]	Quasi-experimental	To deliver education and support to adults with type 2 diabetes mellitus.	Chatbot, web-based, tree-based	App data analytics, surveys (n=150)
Pienkowska et al (2023) [55]	Qualitative	To educate adults living with type 2 diabetes about their condition.	Chatbot, app-based, tree-based	Web-based survey, structured interview (n=8)
Roca et al (2021) [56]	Quasi-experimental	To improve medication adherence in patients with comorbid type 2 diabetes mellitus and depressive disorder.	Chatbot, messaging service, tree-based	Interviews, questionnaires (n=19)
Sagstad et al (2022) [57]	Qualitative	An informational chatbot addition to established care for women with gestational diabetes mellitus.	Chatbot, web-based, AI-based	Chatbot dialogues, data analytics (n=N/A^b^)
Ter Stal et al (2021) [58]	Qualitative	To support users in self-management of chronic diseases in a long-term, daily life setting.	Embodied, app-based, tree-based	Ratings, data analytics, semistructured interviews (n=11)
Tongpeth et al (2018) [59]	Qualitative	To improve patients’ knowledge of, and response to, acute coronary syndrome symptoms.	Embodied, app-based, tree-based	Focus groups, satisfaction questionnaire (n=22)
Tsai et al (2022) [60]	Qualitative	To support patients with chronic kidney disease and manage their condition.	Chatbot, app-based, AI-based	Structured questionnaire, one-on-one interviews (n=26)
Zhang et al (2015) [61]	Qualitative	To counsel patients on their diagnoses and medications specified by a clinician.	Embodied, app-based, unreported	Scales (n=10)

^a^AI: artificial intelligence.

^b^N/A: not available.

### Study Quality

Included studies met most criteria outlined in the relevant Joana Briggs Institute critical appraisal tools with 2 exceptions, Echeazarra et al [48] and Tsai et al [60] (Multimedia Appendix 2). The most common unmet criterion for RCTs was a lack of ensured blindness, for quasi-experimental studies, it was a lack of pre- and postmeasurements, and for qualitative studies, it was unaddressed researcher influence (Multimedia Appendix 2).

Several other criteria relating to ethical considerations and data privacy were also assessed for each study: 14 of 20 studies reported on whether institutional review board approval was received, 2 of 20 studies reported on whether interventions complied with local data protection regulations, 7 of 18 studies reported on user consent procedures (participant consent was not required for 2 of the studies), 0 of 20 studies reported on user data control options, 0 of 6 studies reported whether artificial intelligence (AI) agents used explainable AI, and 0 of 6 studies reported whether efforts were made to address potential biases in AI models. These gaps highlight a critical concern: while conversational agents in health care handle sensitive personal data and influence health decisions, most studies (18/20) lacked comprehensive ethical frameworks. This represents a significant research gap, requiring urgent attention, as AI-driven health interventions become more prevalent.

### Domains of Design Features

In total, 5 domains and 13 subdomains pertaining to design features that impact user engagement with conversational agent–enabled interventions emerged (Table 2). The 5 domains (and corresponding subdomains) were communication style (anthropomorphism, multiple choice, pacing, and redundancy), functionality (miscellaneous, embodiment, and personalization), accessibility (platforms and tutorials), visual appearance (anthropomorphism and gender), and personality (anthropomorphism and mentorship). Data that reported on design features or user engagement separately were used to identify proposed features and research gaps. Some studies did not directly address the impact of design features on user engagement but were useful in identifying research gaps [52,53,61]. Key findings are reported here; for comprehensive descriptions of how design features impacted engagement, refer to Multimedia Appendix 3 [35-38,46-61].

**Table 2:** Domains and subdomains of conversational agents and the studies that reported their impacts on engagement.

Domains and subdomains		Reference
**Communication style**		
	Anthropomorphism	[35,36,38,47,58]
	Multiple choice	[35,54-56,58,60]
	Pacing	[38,47-49,54,56,58,59]
	Redundancy	[47,51,54,56,57]
**Functionality**		
	Miscellaneous	[50,54,56,59]
	Embodiment	[36,37]
	Personalization	[51,58]
**Accessibility**		
	Platforms	[35,38,48,60]
	Tutorials	[35,38,46,49,56]
**Visual appearance**		
	Anthropomorphism	[36,46,55,58,59]
	Gender	[58]
**Personality**		
	Anthropomorphism	[36]
	Mentorship	[36,51,55,58,59]

### Communication Style

Communication style describes how a conversational agent interacts with the user. A natural flow of conversation was credited by most participants, with a favorable view of the intervention by Cheng et al [35] as the reason for its high usability. Additionally, voice quality, including factors like tone, cadence, and pronunciation, impacted user satisfaction and the perceived anthropomorphism of the agent [36,47]. Users appreciated being able to ask agents about information already provided by the health care service or the internet, “You can bring up information on [atrial fibrillation] on the internet and read most of the same stuff on your own, but with Tanya you have a structured presentation that you’re led through, so you end up getting the information you should be getting” [51,57]. Many users found multiple choice dialogues limiting and expressed frustration, leading to early disengagement [35,56,58]. Inundating users with “too much reading” or “repetitive” content caused burnout or disinterest [38,47,58]. Preferences regarding communication frequency varied among users, with a tendency for users preferring weekly communications [49]. Studies using both chatbots and embodied agents contributed similar amounts of evidence on engagement outcomes for the accessibility domain. Most engagement outcomes reported in studies using chatbots related to design features within the communication style domain.

### Functionality

Functionality describes the practical capabilities of the agent. A range of functions improved user engagement, including gamification elements, agent-enabled quizzes, and weather forecasting [36,56,59]. There were conflicting reports pertaining to voice assistant functionality conversational agents, with 1 study showing no effect, and another had users reporting voice added depth to communications “instead of reading it, you’re hearing it” [36,37]. Agents that did not account for user context and time constraints led to user dissatisfaction, with irrelevant content reducing user engagement, “When I have to go to work, I do not have time to watch a 15-minute video” [51,58]. Most engagement outcomes for design features within the functionality domain were provided by studies using embodied agents.

### Accessibility

Accessibility describes the measures taken to ensure that different types of users can interact with the agent. Technical challenges and a lack of familiarity with technology were issues among some users [35,46]. Low digital literacy resulted in various barriers to engaging with interventions, like users accidentally disabling the sound of reminders and then being unable to unmute them [38,56]. Even with thorough out-patient tutorials, certain demographics, such as older patients with limited digital experience, still faced challenges using the technology independently at home [49]. Older patients tended to prefer voice-activated platforms like Google Home over smartphones for conversational agents, but they faced challenges like physical inconvenience, language barriers, and technology support needs [35]. A platform that minimized installation requirements, by hosting their agent on an app users already had downloaded, was appreciated by users [60]. Studies using chatbots and embodied agents contributed similar amounts of evidence on engagement outcomes for the accessibility domain.

### Visual Appearance

Visual appearance describes the physical design and visual representation of the agent. While some users favored anthropomorphic agents due to their perceived credibility, others leaned toward cartoon figures for a more “fun” and user-friendly experience [36]. A photo-realistic design, especially one that fits the context, such as a nurse in a health care setting, was reported to make interactions feel more genuine and personal [58]. Ensuring human-like facial expressions and gestures was important for avoiding negative impacts to the user experience [36,46,59]. Sylvia’s identity as a woman provoked mixed reactions, with one user calling her a “stupid woman,” while another liked her identity as they “hated” listening to men [58]. Only studies using embodied agents contributed evidence on engagement outcomes for the visual appearance domain.

### Personality

Personality describes the character and demeanor that the agent embodies. Incorporating human-like mannerisms increased users’ engagement with Laura, but her backstory led to frustration for some users, who felt that it was excessive and a waste of time, as Laura was not real [36]. Several users who perceived Laura to be authoritative developed feelings of guilt, leading some to temporarily cease communications and others to consider reporting only their best readings [36]. Whereas users who viewed Laura as friendly and supportive tended to be more engaged during communications [36]. Users explained that because Tanya and Nurse Cora did not say “very drastic things,” they felt encouraged to take manageable actions in their daily lives to “do something simple daily” [51,59]. One user expressed a preference for an authoritative agent over a friendly agent, “She could be your girl next door...If I have medical complaints, I prefer an authority to explain what to do or not to do” [58]. Only studies using embodied agents contributed evidence on engagement outcomes for the visual appearance domain.

### Proposed Features

Proposed features describe features suggested by users that they felt would enhance their impression of or engagement with the intervention and represent ideas for further exploration. While in-app navigation was intuitive for most users of Nurse Cora, some users indicated that they would have liked instructions on how to navigate the app containing Nurse Cora at the beginning of the trial [59]. Multiple-choice only functionality was frequently a point of frustration for users of conversational agent–enabled interventions, making one participant “consider [them]self a passive participant” in the conversation [35,38,47,54-56,58,60]. Molly users suggested that she omit punctuation when speaking and incorporate voice recognition [49]. Users of Nurse Cora wanted her to emphasize keywords using larger font sizes and to provide the option to repeat dialogues [59]. Vitória users suggested developing a “strategy to remind users to use the app” and offering specialized content such as “recipes for people with diabetes” [47]. Sylvia users desired more follow-up and checks on prior interactions, “Did you read that? Did you do this?” [58]. This sentiment was also expressed by Nurse Cora users who desired more feedback during the quizzes [59].

### Research Gaps

Research gaps were found in the way engagement of conversational agent–enabled interventions tended to be reported. Conversational agent–enabled interventions were frequently rated in terms of their engagement, but the rationale behind these ratings was not explored except for the study by Cheng et al [35,38,46,48,50-54,56,58,59,61]. For this study, a natural flow of conversation was credited by most participants, with a favorable view of the app as the reason for its high usability [35]. Additionally, differing levels of engagement, trust, or perceived care based on ethnicity, medication regimes, age, education, and computer literacy were identified but not explained [37,61].

## Discussion

### Principal Findings

This systematic review synthesized evidence pertaining to conversational agent–enabled intervention design features and their impacts on the engagement of people self-managing CMD. Integrating redundancy and anthropomorphism to increase user autonomy and investment, respectively, emerged as 2 strategies for improving engagement relevant to all 5 emergent domains. However, care must be taken to avoid inundating users with excessive redundancy or anthropomorphism, as these were common frustrations among users across studies. The findings from this review can be applied to optimizing the development and evaluation of conversational agent–enabled interventions, facilitating more engaging and effective strategies for supporting people self-managing CMDs. Additionally, this review establishes a foundation for upcoming research by identifying research gaps and synthesizing an array of innovative design features suggested by users (Textbox 1).

**Design checklist.**

- Provide multiple input and output modalities so users can use those that match their preferences or needs. Within each modality, allow users to customize design features, such as increasing text sizes or toggling agent accents.
- Offer a “Skip” button so users can bypass dialogues they perceive as frustrating and time-consuming, such as character backstories.
- Ensure sensitivity to how much time users have for a session and adapt content accordingly.
- Provide expandable dialogue options that users can ignore to determine the pacing of conversations.
- Avatar choices should reflect agent functions, users expect serious activities to be delivered by more realistic avatars, and fun activities to be delivered by more cartoonish avatars.
- Provide multiple options for users to learn how to use agent features, such as in-person, in-app, or helplines.

Redundant design features are those that offer additional input options and delivery channels of content. Redundancy is relevant to all 5 emergent domains, as it can be incorporated into modes of communication, offering overlapping functions and inputs and supporting adaptive interaction styles and various other aspects of intervention design. By offering redundant design features, users are empowered to interact with interventions on their own terms, thereby improving sustained engagement. For example, Roca et al [56] reported that one patient was unable to respond to medication reminders, confirming they had taken their medicine because the agent did not understand their messages. In total, the authors recorded 34 such misunderstood interactions between this patient and the agent. Despite this issue, the patient managed to manually log their medication adherence, illustrating the critical role of redundancy within interaction options [56]. A lack of redundancy in accessibility led to problems for Gingele et al [49], who found that despite providing comprehensive outpatient tutorials, users encountered significant challenges when attempting to use conversational agent–enabled interventions independently at home. These challenges could be addressed by offering additional tutorial options like helplines, outpatient sessions, and in-app guides [49]. Despite the benefits of redundancy in promoting user autonomy, empirical evidence identified within this review also underscored the importance of ensuring that users are not inundated with irrelevant content [38,47,58]. For example, Ter Stal et al [58] highlighted instances where participants felt overwhelmed by excessive and inappropriate communication frequency, leading to frustration and disengagement, “When I have to go to work, I do not have time to watch a 15-minute video.” Furthermore, Epalte et al [38] reported a participant complaining of “too much reading,” and Balsa et al [47] reported users identifying information overload and excessive queries as areas for improvement. Similarly, Pop-Eleches et al [63] found that weekly SMS text message reminders significantly improved antiretroviral adherence, whereas daily reminders did not.

The assertion that offering redundant design features improves user engagement by enabling flexibility in interaction aligns with findings grounded in self-determination theory from a tutorial paper by Yardley et al [26]. Self-determination theory posits that engagement can be improved by supporting user autonomy, competence, and relatedness [64]. By providing redundancy in options and content, users are empowered to choose their interaction methods, access alternative options, and find content that feels personally relevant, thereby supporting user autonomy, competence, and relatedness [26]. An alternative explanation for why redundant design features improved user engagement is that they create a more consistent user environment where repeated exposures to similar content can make new information more familiar and accessible [65]. This explanation links into the concept of omnichannel engagement, describing uniformity between content channels, which is elaborated in a viewpoint paper by Blasiak et al [65]. There were several examples where users appreciated interventions providing content, which they could access elsewhere [51,56,57]. For example, Sagstad et al [57] and Guhl et al [51] reported that users appreciated accessing information through the conversational agents within the interventions, which they knew they could access on the web or from their doctor [51,57]. Furthermore, all participants within the study by Roca et al [56] gave an affirmative response to the statement, “From a certain moment, the virtual assistant began to give the weather forecast. Has this helped you to use the virtual assistant more frequently?”

Anthropomorphic design features are those that contribute to users attributing human traits, emotions, or intentions to the intervention. Anthropomorphism is relevant to all 5 emergent domains, as it can be incorporated into the conversational style, personality, visual design, and various other aspects of intervention design*.* By offering anthropomorphic design features, users can become more invested in interventions, thereby improving sustained engagement. For example, Ter Stal et al [58] reported a participant stating that the realistic depiction of the conversational agent made the interaction more personal. Similarly, Baptista et al [36] reported user preference for Laura’s human character over a cartoon character, “I’m not sure I would have given the same level of credibility to, for example, a dog or a cat or something like that.” Furthermore, a common point of frustration among users across studies was with conversational agents which were “not human enough”; these issues pertained to the speech, mannerisms, gestures, and expressions of conversational agents [36,46,59]. Despite the benefits of anthropomorphism in promoting user investment, empirical evidence identified within this review also identified instances where efforts toward this goal were excessive or came at the expense of other functionalities [36,58]. An example of an excessive pursuit of anthropomorphism was identified by Baptista et al [36], who observed that providing a backstory for their conversational agent led to frustration for some users. Here, a backstory feature in the personality domain, when projected through an anthropomorphized avatar in the visual appearance domain, resulted in this avatar increasing frustration rather than investment with the intervention itself. Furthermore, Ter Stal et al [58] reported that most participants expressed that they were not interested in the small talk from their conversational agent.

The contention that offering anthropomorphic design features improves user engagement by promoting user investment in interactions aligns with the computers are social actors framework. The computers are social actors framework posits that engagement can be improved by supporting user investment by exploiting users’ innate social expectations to make interactions more meaningful [66]. However, the computers are social actors framework fails to explain why excessive anthropomorphism leads to user frustration. Ruslana and Ning [67] identified excessive anthropomorphism as problematic because it caused confusion for users. However, they also suggested an alternative explanation for its positive effect on engagement: anthropomorphic design features may make interventions feel more approachable and less formal. This suggests that developers should carefully consider the function of anthropomorphism and be willing to forego it if simpler methods can achieve more approachable interventions. For example, Baptista et al [36] observed that certain users favored cartoon figures over anthropomorphic agents, as they were more “fun.” Similarly, Thunström et al [68] found that a mental health chatbot was rated more usable than an anthropomorphic embodied conversational agent, though the usability scale used in this study did not have the fidelity to attribute this to a specific feature differing between the agents.

### Limitations

This systematic review faced limitations primarily due to the heterogeneity of included interventions, outcomes, and study types. In terms of interventions, the field of conversational agent–enabled interventions is rapidly evolving, with new technologies and platforms emerging regularly. This resulted in a wide variety of conversational agent–enabled interventions included in the review, ranging from basic chatbots with multiple choice responses to embodied conversational agents. In terms of outcomes, engagement in digital health lacks a standardized definition, leading to different evaluations and operationalizations between studies. For example, some studies equated it directly with use, while others conceptualized it as a composite of behavioral, cognitive, and affective components. The diversity in how engagement was defined and measured across studies limited the synthesis of findings, pointing toward the need for a standardized operationalization of engagement within digital health contexts, to enable more precise and comparable measurements. In terms of study types, papers included in this review were primarily qualitative or quasi-experimental studies, restricting our ability to compare design features with quantitative user engagement metrics through meta-analysis. Together, these heterogeneities limited our ability to compare results across studies and draw definitive conclusions about the impacts of certain design features on engagement, so findings should be interpreted as directional rather than quantitative. Furthermore, studies were conducted in home-based self-management contexts, which are not equally applicable across other health care contexts, such as hospitals and primary care settings. However, the review proceeded with these limitations as more specific inclusion criteria for interventions, outcomes, and study types would have reduced the sensitivity of this systematic review in identifying insights pertaining to design features and their impacts on engagement and in identifying research gaps and opportunities for further research. To address these limitations, we recommend that future systematic reviews focus on specific agent types (eg, embodied agents only) with standardized engagement definitions and conduct individual participant data meta-analyses when sufficient high-quality RCTs become available.

Areas for further research were identified based on the proposed features, research gaps, and limitations identified within this systematic review. A research gap that was consistently identified across studies was that engagement ratings from participants were frequently collected, though the qualitative processes underlying these ratings were rarely explored, hindering a deeper understanding of engagement. For example, Echeazarra et al [48] asked users to rate ease of use and usefulness of their intervention on a Likert scale but did not ask users to explain which features contributed to these ratings. The study by Cheng et al [35] provided a protocol for addressing this research gap, and users were prompted to explain which features contributed to their ratings. Another research gap was that clear and unexplained disparities in how different demographics engage with conversational agent–enabled interventions were identified in multiple studies [37,61]. For example, differing levels of engagement, trust, or perceived care were observed based on ethnicity, medication regimes, age, education, and computer literacy [37,61]. Further research could investigate how design features could be tailored for these different groups, such as using co-design methodologies to explore the role of redundancy in improving engagement of people with poor computer literacy through emphasizing multiple interaction modalities. We recommend factorial RCTs testing specific combinations of design features (eg, 2×2 design testing high or low redundancy×high or low anthropomorphism) across diverse populations, with standardized engagement metrics including both behavioral analytics and qualitative experience measures.

### Conclusions

This systematic review provides key insights and rationales for developing conversational agent–enabled interventions for people self-managing CMD. However, because the included studies varied widely in agent modalities and engagement definitions and were largely qualitative, the generalizability of these insights is limited. Designing conversational agent–enabled interventions requires a balance of redundancy and personalization to support user autonomy without overwhelming users. Additionally, a balance between anthropomorphism and functionality is required to support user investment without frustrating users. Maintaining these balances is a key to improving engagement with conversational agent–enabled interventions and supporting people with CMD to self-manage their condition.

## Appendix

Multimedia Appendix 1 PRISMA checklist.

Multimedia Appendix 2 The complete search strategy.

Multimedia Appendix 3 Comprehensive descriptions of how design features impacted engagement.

## Abbreviations

AI: artificial intelligence
CMD: cardiometabolic disease
PRISMA: Preferred Reporting Items for Systematic Reviews and Meta-Analyses
RCT: randomized controlled trial
