# Supplementary material for: Engagement With Conversational Agent–Enabled Interventions in Cardiometabolic Disease Self-Management: Systematic Review
Source: JMIR Mhealth Uhealth. 2025 Sep 18;13:e67913. doi: 10.2196/67913 (PMC12491886; doi:10.2196/67913)
Supplement: Multimedia Appendix 2 [file mhealth_v13i1e67913_app2.docx]

**Search strategy**

“Cardiovascular Diseases”[MeSH Terms] OR metabolic OR diabetes OR diabetic OR "metabolic syndrome" OR dyslipidemia OR dyslipidaemia OR "insulin resistance" OR "glucose intolerance" OR "impaired glucose" OR "high cholesterol" OR hyperlipidemia OR hyperlipidaemia OR hypercholesterolemia OR hypertriglyceridemia OR obesity

AND

"conversational agent*" OR chatbot* OR "virtual agent" OR "virtual assistant" OR "automated speech recognition" OR "relational agent" OR "embodied conversational agent" OR "virtual assistan*" OR "dialog* system" OR "digital assistan*" OR avatar*

AND

accept* OR perceived OR perception OR useful OR inten* OR use OR using OR attitude OR satisfy* OR producti* OR competen* OR adopt* OR resist* OR influence OR social OR rated OR rating OR adher*

**Table 1:** Study design and population

| **Author Year** | **Study design** | **Data collection methods** | **Sample size** | **Sociodemographics** | **Inclusion criteria** | **Exclusion criteria** |
| --- | --- | --- | --- | --- | --- | --- |
| Apergi et al 2021 (37) | Quasi-experimental | Surveys, questionnaires, analytics | 30 | Mean age (SD): 56.5 (12.1) 10/27 (37%) females Black: 63% | All participants had at some point in the past either been admitted to the MedStar Washington Hospital Center for HF or had been seen in a MedStar Heart Failure Clinic for HF. Furthermore, the patients were required to be aged 18 years or older and live in a house with Wi-Fi access. | The patients could not participate in the studies if they had had a heart transplant or if they had a ventricular assist device |
| Balsa et al 2019 (46) | Qualitative | Self-administered questionnaire | 28 (19 healthcare professionals and 9 adults with T2D) | Nineteen healthcare professionals (12 nurses and 6 physicians; mean 46.22, min 25 max 63) plus nine adults with T2D accepted to participate. Older people’s age ranged between 66 and 89 years (mean 71). | Nurses, physicians and older people with T2D were purposively sampled within five primary care units of the Portuguese National Health Service. | NA |
| Balsa et al 2020 (47) | Qualitative | Questionnaire, open questions, diaries, digital notes, telephone follow-ups | 20 (11 with T2D) | Feature of end users; Gender; Female= 27.3% (n=3) and Male=88.9% (n=8), Mean age (years) 70.91, [max: 80, min: 67], Education college degree is 63.6% (n=7) and secondary education 36.4% (n=4), Mean experience in the use of technology usage (years) 6.09 [max: 20, min:0]   Feature of Experts Gender Female is 72.2% (n=8) and Male is 11.1% (n=1), Mean age (years) is 54.33 [max: 63, min: 40], Mean professional experience (years) is 31.7 [max: 40,min: 19]. | Inclusion criteria were having T2D, age equal or above 65 years, being able to speak and write in Portuguese, and being a frequent user of digital technology (e.g. possessing a smartphone or using social media or a computer). The sample of experts included nine academic nurses with expertise in community health and elderly care, recruited in the Lisbon School of Nursing | NA |
| Baptista et al 2020 (36) | Quasi-experimental | Web-based surveys, Interview | 93 | Female: 44/93 (47%) Mean age (SD): 55 (10) | "Adults with T2D registered on the National Diabetes Services Scheme (NDSS) database; willing to be contacted about research; and living in New South Wales, Queensland, Victoria, and Western Australia." | NA |
| Cheng et al 2018 (35) | Qualitative | Feature based comparison with other mobile applications, subjective tests for qualitative assessment of effectiveness and satisfaction, gathering feedback from elder-care experts and potential users. | 10 | NA | the test was conducted on a group of 10 willing elderly participants, in which each participant was asked to answer a series of survey questions that were similarly structured to the depression screening survey on the Healthy Coping application. | NA |
| Echeazarra et al 2021 (48) | RCT | -BP measurements (Holter device) -Knowledge and skills checklist -Satisfaction survey | 112 patients | -Mean age (Range): 52.1 (21.0-87.0) -47/112 (42%) Women | -Patients attending the Nephrology outpatient clinic (CCEE) of the Araba University Hospital (HUA), with a diagnosis of hypertension or suspected primary or secondary hypertension. They must be at least 18 years old and have been previously instructed to perform BP checks at home with an information sheet available at the outpatient hospital. -Patients must have an approved and calibrated tensiometer and a smartphone with Internet connection at home, and sufficient ability to send messages via WhatsApp or Telegram. | -Previous diagnosis of severe psychiatric disorder. -Severe illness suggesting a life expectancy of less than 6 months or high probability of needing haemodialysis or transplantation in less than 3 months. -Severe clotting disorders that can cause bruising if frequent BP measurements are performed. -Motor or visual disability that makes it difficult to perform self-measurements at home |
| Epalte et al 2023 (38) | Qualitative | Semi-structured interviews | 12 | Mean age (range): 57 years (40-82 years) 7 females, 5 males. | (1) at least 18 years of age, (2) no previous record of ischaemic or haemorrhagic stroke; (3) level of knowledge of Latvian language to understand and answer the interview questions; (4) minimum score of 18 on Montreal Cognitive Assessment (MoCa); (5) functionally able to use a tablet. | A recurrent stroke, psychiatric or any other comorbidity that might affect participation |
| Gingele et al 2022 (49) | Qualitative | Health questionnaires, system usability scale, self-developed patient satisfaction scale, general feedback form. | 37 heart failure patients | Mean age (SD): 67 years (10.9 years) Female: 30% | All HF patientsâ‰¥18 years of age, diagnosed with HF, irrespective of left ventricular ejection fraction, were eligible for inclusion. | NA |
| Gong et al 2020 (50) | RCT | Data analytics, blood test, Assessment of Quality of Life-8D scale, Problem Areas in Diabetes scale, process evaluation and, clinical measurements of body weight | 187 | Female: 41.7% Mean age (SD): 57 (10) years | Adults (aged â‰¥18 years) diagnosed with T2DM, registered with the National Diabetes Service Scheme (NDSS) for less than 10 years, with basic English language skills, who had access to an internet-enabled smart device with an up-to-date operating system (at least iOS 8.0 for Apple and 4.2 for Android). | Pregnant or planning to become pregnant, had severe comorbid conditions that would compromise their participation, or did not have stable doses of diabetes-related medication over the previous 4 weeks or more. |
| Guhl et al 2020 (51) | Quasi-experimental | Measures, self-reports | 120 | Female, n (%): 62 (51.7%) Mean age (SD): 72.1 years (9.10) | Inclusion criteria were (1) age â‰¥18 years, (2) history of chronic AF, (3) prescribed oral anticoagulation for stroke prevention secondary to AF, and (4) English-speaking sufficient to use a smartphone-based relational agent as ascertained by the study screener. | Participants were excluded from this pilot trial if they had AF deemed attributable to a non-cardiac cause, had undergone cardiothoracic or thoracic surgery within 30 days of evaluation, were unable to use the smartphone apps after training, had a life expectancy of <12 months as identified by a concurrent diagnosis (such as malignancy), or by determination of the research team for not being able to participate in the informed consent process. |
| Kimani et al 2016 (52) | Quasi-experimental | Data analytics, self-report scale and semi-structured interview | 16 | Female: n=5 (31.25%) Mean or median? age (range): 40 (20-58) years | We recruited participants from Boston University Medical Center for our pilot study. Participants were required to be 18 years of age or older; English speaking, in possession of an iPhone, and able to independently consent to participate in the project. | NA |
| Magnani et al 2017 (53) | Quasi-experimental | Qualitative interviews, and questionnaire | 31 | Mean age (SD): 68 (11) Women: n=12 (39%) | Adult (age â‰¥18), a diagnosis of nonvalvular AF as ascertained by review of the electronic health record, CHA2 DS2-VASc score â‰¥2, and receiving oral anticoagulation. | Having an identified extracardiac cause of AF (such as sepsis or thyroid disease), as the management of AF in such context may differ based upon the underlying etiology; inability to provide accurate three-word recall; inability to provide informed consent; or being non-English speaking. |
| Nassar et al 2023 (54) | Quasi-experimental | App data analytics, surveys, patient data from hospital records (Glycemic outcomes (A1C)). | 150 | Mean age (SD): 58.3 (10.6) years, 90/150 participants were female. | -T2DM on the electronic medical record Problems List -Patient of a system provider (n=4)  -A1C of 8-8.9% OR recent completion of the systems intensive diabetes care management program -Access to a smart phone and/or email | N/A |
| Pienkowska et al 2023 (55) | Qualitative | Web-based survey and structured interview | 8 | -Mean age (SD): 56 (13.79) years  -5 men and 3 women | -Over 20 years old  -Person with T2D  -English speaker  -Own a computer, tablet device, or smartphone | -Person with T1D  -Carer for person living with T1D  -Unable to provide consent |
| Roca et al 2021 (56) | Quasi-experimental | Blood tests, medical possession ratio, interviews, and questionnaires | 14 patients and 5 healthcare professionals -1 patient did not interact with the virtual agent and did not complete the trial | Mean age (SD): 63.8 (9.1) years Female: n=9/13 (69%) | For patients  -Patients with type 2 diabetes and depressive disorder who were 18 years old or older. -Patients must have regular appointments with the nurses. -Patients need to take medication every day. -Patients have poor medication adherence. Poor adherence is measured with the level of the medication possession ratio (MPR). MPR is the division between the number of drug units prescribed for a specific period divided by the number of days. The MPR value is capped at 100%. A presence/absence of medication adherence is calculated with a binary variable. When the MPR value is below 80%, the medication adherence is considered as absence. -Patients can read and understand Spanish. -Patients have the ability to write a message in a messaging platform. -Patients need to have a smartphone with Android or iOS, and they need to have access to the internet in their smartphones. | For patients -Cognitive, visual, or physical impairments that would interfere with the use of the virtual assistant. |
| Sagstad et al 2022 (57) | Qualitative | Chatbot dialogues, data analytics | NA | NA | NA | NA |
| ter Stal et al 2021 (58) | Qualitative | Ratings, data analytics, and semi-structured interviews | 11 | -Median (range): 70 (49-83) years -Female: n=4 (36%) | (1) had a clinical diagnosis of both COPD and CHF; (2) had at least two COPD or CHF exacerbations or at least one hospitalization for COPD or CHF in the 2 years preceding study entry; (3) were at least one week after prednisolone/antibiotics/furosemide course and hospitalization and at least four weeks after rehabilitation; (4) were at least 40 years of age; (5) were able to understand and read the Dutch language; (6) were able to use a smartphone, tablet, or PC; and (7) provided written informed consent prior to participation. | (1) had terminal cancer or were at the end stage of another serious disease, (2) had another serious lung disease, (3) expected cardiovascular intervention within 3 months, (4) were enrolled in randomized controlled trials or a trial with study medication, (5) were waiting for a heart or lung transplantation, and (6) received renal dialysis. |
| Tongpeth et al 2018 (59) | Qualitative | Focus groups, ACS Response Index score, Satisfaction questionnaire | 10 consumers from the cardiac rehabilitation unit and 12 clinical and research experts | Mean age (SD): 52.2 (10.37) years Female: n=1 (10%) | Had been admitted for any cardiac diagnosis in which chest pain or other heart attack symptoms had been experienced, had not received education prior to reviewing the app, were clinically stable, had normal cognitive function, had sufficient English language ability to communicate and follow the study procedures, and were able to give informed consent. | NA |
| Tsai et al 2022 (60) | Qualitative | Literature review, structured questionnaire, one-on-one interviews | Phase 1: 9 patients and 2 clinicians Phase 3: 15 patients | Age ranges (n): 30-40 (5), 40-50 (3), 50-60 (1) | Chronic kidney disease diagnosed by doctors with a glomerular filtration rate less than 60 mL/min/1.73 m^2, the course of disease over 3 months, and with experience using smartphones as subjects. | NA |
| Zhang et al 2015 (61) | Qualitative | Scales | 10 patients | Mean age (range): 85.89 (70-94) Female (%): n=2 (20%) | Local geriatric patients from the Xin Hua Hospital in China | NA |

**Table 2:** Intervention Description

| **Author Year** | **Name of conversational agent** | **Conversational agent role** | **Conversational agent type** | **Conversational agent platform** | **Intervention duration** | **Intervention frequency** | **Comparator description** |
| --- | --- | --- | --- | --- | --- | --- | --- |
| Apergi et al 2021 (37) | NA | To ask the patients the same series of questions related to their HF treatment and symptoms and provide feedback. | Embodied conversational agent | App-based | 90 days | Participant discretion | An augmented version of Alexa, delivered via an Echo Dot device, which was capable of delivering a voice-activated survey that could give feedback. |
| Balsa et al 2019 (46) | Vitória | Assisting older people with Type 2 Diabetes Mellitus (T2D) in medication adherence and lifestyle changes. | Embodied conversational agent | App-based | Single face-to-face session | Once | NA |
| Balsa et al 2020 (47) | Vitória | Designed as a relational agent, the virtual assistant has the role of supporting older people with Type 2 Diabetes Mellitus (T2D) in medication adherence and lifestyle changes. | Embodied conversational agent | App-based | End-users were asked to undertake a trial period of 26 days at home, guided by a list of suggested tasks, which included talking to Vitoria daily.  Experts were asked to test the prototype independently over a shorter period (8 to 10 days), by means of multiple interactions with Vitoria on the same day. | Participant discretion (recommended to use intervention daily) | NA |
| Baptista et al 2020 (36) | Laura | "To deliver self-management education and support to adults with T2D." | Embodied conversational agent | App-based | 12 months | Mean total interactions with Laura (SD): 18 (15) | NA |
| Cheng et al 2018 (35) | NA (Google Home's voice interface) | To allow for a less cumbersome way for geriatric T2DM patients to effectively adhere to DSM guidelines. | Embodied conversational agent | Google Home and APLAI platform | Workshop of undisclosed length of time | Single Workshop | NA |
| Echeazarra et al 2021 (48) | TensioBot | To help patients with hypertension to self-monitor their blood pressure | Chatbot | messaging service (Telegram and WhatsApp). | Intervention subjects were contacted by TensioBot 7 days before their first medical appointment of the RCT. TensioBot use continued until a second medical appointment of the RCT, it is unclear how long this period of time went, though the RCT lasted 2 years. | BP measuring prompt twice a day.  Daily tips regarding good BP measurement practice.  Intervention frequency is toggleable by the patient. | Each control patient attending their first medical appointment receives a written procedure on how to self-monitor their BP. The nurse assesses the patients’ knowledge and skills on BP self-monitoring using a checklist and ensures that the patient knows when their next medical appointment is and when to perform BP measurements with a Holter device. |
| Epalte et al 2023 (38) | Vigo | To counsel, educate, and train the stroke patient and patient’s family on stroke, rehabilitation, care, and other related issues | Chatbot | App-based | Average (range): 36 days (6-49 days) | Participant discretion | NA |
| Gingele et al 2022 (49) | Molly | It regularly provides online, written questionnaires in the Dutch language based on common HF signs and symptoms to evaluate patients’ health status. Educational sessions, including written information on the HF syndrome and HF care, are provided as well. Patients are able to communicate with HF nurses by sending and receiving messages and notifications via the HF coach. | Embodied conversational agent | App-based | 90 days | patients received health questionnaires on a daily basis during the first month. Thereafter, questionnaires were provided once a week for 2 months, unless increasing symptoms required shorter intervals. | NA |
| Gong et al 2020 (50) | Laura | To provide more accessible and engaging self-management support, monitoring, and coaching to adults with T2DM in Australia. | Embodied conversational agent | App-based | 12 months | Participant discretion | Participants in the control arm were encouraged to continue their routine diabetes self-care, including access to health care services, resources accessed via NDSS, and the diabetes not-for-profit organizations in their states. They received a quarterly project newsletter to maintain their interest in the study. Following the 12-month data collection, participants in the control arm received access to the MDC program if they wished. |
| Guhl et al 2020 (51) | Tanya | To augment patient-centered health care by providing health education, monitoring, and problem-solving for users. | Embodied conversational agent | App-based | 30-days | Median of 18 (interquartile range 19) conversations with the relational agent over duration of the intervention. | Usual care |
| Kimani et al 2016 (52) | NA | During the first week of the intervention, the counselor prioritizes education in its dialog, explaining what AF is, how to effectively use the heart rhythm monitor, and describing common symptoms associated with the condition. Over long-term use, the agent promotes adherence to daily heart rhythm monitor readings. | Embodied conversational agent | App-based | One week | Participant discretion | N/A |
| Magnani et al 2017 (53) | Tanya | Education, motivation, and monitoring (symptoms and adherence) | Embodied conversational agent | App-based | 30 days | Participant discretion | N/A |
| Nassar et al 2023 (54) | MedStar Health | Education and support | Chatbot | App-based | At least 6 weeks | -Participants may contact the chatbot on demand. But there are certain times when the chatbot will contact them.  -Initial session on day of enrolment  -Subsequent session a week later on a day of the participants choosing  -Followed by four more weekly sessions  -Then regular weekly/fortnightly sessions (participant discretion) | N/A |
| Pienkowska et al 2023 (55) | N/A | Diabetes education | Chatbot | App-based | Single session | Single session | N/A |
| Roca et al 2021 (56) | N/A | To improve medication adherence in patients with comorbid type 2 diabetes mellitus and depressive disorder. | Chatbot | Messaging service | 9 months | Participant discretion | N/A |
| Sagstad et al 2022 (57) | Dina | An informational chatbot addition to established care for women with gestational diabetes mellitus | Chatbot | Integrated into the Norway's national digital health platform | Dialogues were collected from the chatbot’s log and platform over 20 weeks (from week 41 to 48 in 2018 and from week 47 in 2019 to week 6 in 2020). | Participant discretion | N/A |
| ter Stal et al 2021 (58) | Sylvia | To support users in self-management of chronic diseases in a long-term, daily life setting | Embodied conversational agent | App-based | 4 months. | Participant discretion | N/A |
| Tongpeth et al 2018 (59) | Nurse Cora | To improve patients’ knowledge of, and response to, acute coronary syndrome symptoms. | Embodied conversational agent | App-based | Single session | Single session | NA |
| Tsai et al 2022 (60) | NA | To support patients with chronic kidney disease manage their condition | Chatbot | App-based | One week | Participant discretion, recommended daily | NA |
| Zhang et al 2015 (61) | NA | The agent counsels patients on their diagnoses and medications specified by a clinician, as well as increasing physical activity, improving diet, decreasing stress, and motivating them to be more involved and proactive in their own care. | Embodied conversational agents | App-based | One session | A single counselling session with a virtual agent | 4 patients had a counselling session with a human doctor |

**Table 3:** Quality assessment of Randomized controlled studies

|  | **Gong 2020** (50) | **Echeazarra 2021** (48) |
| --- | --- | --- |
| True Randomization Used | Yes | Yes |
| Allocation Concealment Achieved | No | No |
| Baseline Similarity Confirmed | Yes | Unclear |
| Participants Blindness Maintained | No | No |
| Deliverers' Blindness Ensured | No | Unclear |
| Identical Treatment Outside Intervention | Yes | No |
| Assessors' Blindness Ensured | No | Unclear |
| Consistent Outcome Measurement | Yes | Yes |
| Reliable Outcome Measurement | Yes | Yes |
| Complete Follow-up Analysis | Yes | Yes |
| Analysis By Randomization | Yes | Yes |
| Appropriate Statistical Analysis | Yes | Yes |
| Trial Design Justified | Yes | Yes |

**Table 4:** Quality assessment of quasi-experimental studies

|  | **Baptista 2020** (36) | **Guhl 2020** (51) | **Magnani 2017** (53) | **Kimani 2016** (52) | **Apergi 2021** (37) | **Roca 2021** (56) | **Nassar 2023** (54) |
| --- | --- | --- | --- | --- | --- | --- | --- |
| Cause and Effect | Yes | Yes | Yes | Yes | Yes | Yes | Yes |
| Participants Comparably Similar | Not Applicable | Yes | Not Applicable | Not Applicable | Yes | Not Applicable | Not Applicable |
| Similar Treatment Provided | Not Applicable | Yes | Not Applicable | Not Applicable | Yes | Not Applicable | Not Applicable |
| Control Group Present | Not Applicable | Yes | Not Applicable | Not Applicable | Not Applicable | Not Applicable | Not Applicable |
| Pre-Post Measurements | Not Applicable | Yes | Yes | No | No | Yes | Yes |
| Adequate Follow-Up | Yes | Yes | Yes | Yes | Yes | Yes | Yes |
| Outcomes Measured Similarly | Not Applicable | Yes | Not Applicable | Not Applicable | Yes | Not Applicable | Yes |
| Reliable Outcome Measurement | Yes | Yes | Yes | Yes | Yes | Yes | Yes |
| Appropriate Statistical Analysis | Yes | Yes | Yes | Not Applicable | Yes | Yes | Yes |

**Table 5:** Quality assessment of qualitative studies

|  | **Tongpeth 2018** (59) | **Gingele 2023** (49) | **Tsai 2022** (60) | **Cheng 2018** (35) | **Zhang 2015** (61) | **Balsa 2019** (46) |
| --- | --- | --- | --- | --- | --- | --- |
| Philosophy-Methodology Congruity | Yes | No | Yes | Yes | Yes | Yes |
| Methodology-Objective Congruence | Yes | Yes | Yes | Yes | Yes | Yes |
| Data Collection Congruence | Yes | Yes | Yes | Yes | Yes | Yes |
| Analysis Method Congruence | Yes | Yes | No | Yes | Yes | Yes |
| Result Interpretation Congruity | Yes | Yes | No | Yes | Yes | Yes |
| Researcher's Cultural Location | Yes | No | No | Yes | No | Yes |
| Researcher Influence Addressed | Yes | No | No | No | No | No |
| Participants' Representation | Yes | Yes | No | No | No | Not Applicable |
| Ethical Compliance | Yes | Yes | No | No | No | Yes |
| Conclusions' Data Consistency | Yes | Yes | No | Yes | Yes | Yes |

**Table 5 (Continued):** Quality assessment of qualitative studies

|  | **Balsa 2020** (47) | **ter Stal 2021** (58) | **Epalte 2023** (38) | **Sagstad 2022** (57) | **Pienkowska 2023** (55) |
| --- | --- | --- | --- | --- | --- |
| Philosophy-Methodology Congruity | Yes | Yes | Yes | Yes | Yes |
| Methodology-Objective Congruence | Yes | Yes | Yes | Yes | Yes |
| Data Collection Congruence | Yes | Yes | Yes | Yes | Yes |
| Analysis Method Congruence | Yes | Yes | Yes | Yes | Yes |
| Result Interpretation Congruity | Yes | Yes | Yes | Yes | Yes |
| Researcher's Cultural Location | Yes | Yes | Yes | Yes | No |
| Researcher Influence Addressed | No | No | Yes | No | No |
| Participants' Representation | No | Yes | Yes | No | Yes |
| Ethical Compliance | Yes | Yes | Yes | Yes | Yes |
| Conclusions' Data Consistency | Yes | Yes | Yes | Yes | Yes |

**Table 6:** Ethical Considerations and Data Privacy of studies

| **Author Year** | **Conversational agent type** | **Conversational agent platform** | **Complexity (tree-based dialogue systems, LLMs, AI-based, etc)** | **Whether institutional review board approval was received** | **Whether authors state interventions comply with local data protection regulations (GDPR, HIPAA, etc)** | **User consent procedure described** | **User control options (delete data / opt-out) reported** | **Whether AI agents used explainable AI** | **Whether efforts were made to address potential biases in AI models (demographic factors)** |
| --- | --- | --- | --- | --- | --- | --- | --- | --- | --- |
| Apergi et al 2021 (37) | Embodied conversational agent | App-based and Alexa | Tree-based | Unreported | Unreported | Reported | Unreported | NA | NA |
| Balsa et al 2019 (46) | Embodied conversational agent | App-based | Tree-based | Reported | Unreported | Unreported | Unreported | NA | NA |
| Balsa et al 2020 (47) | Embodied conversational agent | App-based | Tree-based | Reported | Unreported | Unreported | Unreported | NA | NA |
| Baptista et al 2020 (36) | Embodied conversational agent | App-based | Tree-based | Reported | Unreported | Unreported | Unreported | NA | NA |
| Cheng et al 2018 (35) | Embodied conversational agent | Google Home and web interface | AI-based | Unreported | Unreported | Unreported | Unreported | Unreported | Unreported |
| Echeazarra et al 2021 (48) | Chatbot | Messaging service | Tree-based | Reported | Unreported | Unreported | Unreported | NA | NA |
| Epalte et al 2023 (38) | Chatbot | App-based | AI-based | Reported | Unreported | Reported | Unreported | Unreported | Unreported |
| Gingele et al 2022 (49) | Embodied conversational agent | App-based | AI-based | Reported | Unreported | Reported | Unreported | Unreported | Unreported |
| Gong et al 2020 (50) | Embodied conversational agent | App-based | Tree-based | Reported | Unreported | Reported | Unreported | NA | NA |
| Guhl et al 2020 (51) | Embodied conversational agent | App-based | Tree-based | Unreported | Unreported | Reported | Unreported | NA | NA |
| Kimani et al 2016 (52) | Embodied conversational agent | App-based | Tree-based | Unreported | Unreported | Unreported | Unreported | NA | NA |
| Magnani et al 2017 (53) | Embodied conversational agent | App-based | Tree-based | Reported | Unreported | Unreported | Unreported | NA | NA |
| Nassar et al 2023 (54) | Chatbot | Web-interface | Tree-based | Reported | Unreported | NA | Unreported | NA | NA |
| Pienkowska et al 2023 (55) | Chatbot | App-based | Tree-based | Reported | Unreported | Reported | Unreported | NA | NA |
| Roca et al 2021 (56) | Chatbot | Messaging service | Tree-based | Reported | Reported | Reported | Unreported | NA | NA |
| Sagstad et al 2022 (57) | Chatbot | Web-interface | AI-based | Reported | Reported | NA | Unreported | Unreported | Unreported |
| ter Stal et al 2021 (58) | Embodied conversational agent | App-based | Tree-based | Reported | Unreported | Unreported | Unreported | NA | NA |
| Tongpeth et al 2018 (59) | Embodied conversational agent | App-based | Tree-based | Reported | Unreported | Unreported | Unreported | NA | NA |
| Tsai et al 2022 (60) | Chatbot | App-based | AI-based | Unreported | Unreported | Unreported | Unreported | Unreported | Unreported |
| Zhang et al 2015 (61) | Embodied conversational agents | App-based | Unreported | Unreported | Unreported | Unreported | Unreported | Unreported | Unreported |
