# Supplementary material for: Engagement With Conversational Agent–Enabled Interventions in Cardiometabolic Disease Self-Management: Systematic Review
Source: JMIR Mhealth Uhealth. 2025 Sep 18;13:e67913. doi: 10.2196/67913 (PMC12491886; doi:10.2196/67913)
Supplement: Multimedia Appendix 3 [file mhealth_v13i1e67913_app3.docx]

**Table 1:** Design features in the domain of Communication Style and sub-domain of Anthropomorphism which impacted user engagement

| **Author Year** | **Feedback** |
| --- | --- |
| Balsa et al 2020 (47) | Users took issue with Vitória’s ‘metallic voice’ and the pronunciation of some words. |
| Balsa et al 2020 (47) | 3/242 comments pertained to language as a positive aspect of Vitória's design, such as “simple language”. |
| Baptista et al 2020 (36) | Discussion about Laura's speech and mannerisms prompted a participant to describe Laura as “‘just another robot’ that they ‘could not connect to’ as she was ‘not human enough’. This was attributed to her “monotone” voice that sounded similar to a ‘mechanised reading mechanism’ with a “strange cadence and inflection”. |
| Cheng et al 2018 (35) | Natural flow of conversation was credited by most participants with a favourable view of the app as the reason for its high usability. |
| Epalte et al 2020 (38) | One participant talked about how the name ‘Vigo’ was “confusing” because of another individual who she knew with that name. |
| ter Stal et al 2021 (58) | A participant said that Sylvia made the app more personal by addressing the user by his or her first name, “It creates a slightly more informal atmosphere, which I always like, I feel a bit more free." |
| ter Stal et al 2021 (58) | Participants expressed disapproval of Sylvia, citing the agent making statements which were too generic, and it seeming like the dialogues were not personalized but instead a series of if-then statements. |
| ter Stal et al 2021 (58) | While five participants stated they were not interested in the small talk which Sylvia communicated, two participants thought that lonely people might be interested. |
|  |  |

**Table 2:** Design features in the domain of Communication Style and sub-domain of Multiple Choice which impacted user engagement

| **Author Year** | **Feedback** |
| --- | --- |
| Cheng et al 2018 (35) | Restricted answer choices were credited by most participants with a negative view of the app as a reason for their view. |
| Roca et al 2021 (56) | The agent used a menu system of dialogue, wherein patients responded with a number corresponding to a multiple-choice option offered by the agent. Patients found this communication style inconvenient and had difficulty being understood by the agent. Most of the patients that stopped using the virtual assistant did so at the beginning, upon first exposure to this communication style. |
| ter Stal et al 2021 (58) | Two participants stated they would like to be able to type a question and receive a personalized answer rather than select a multi-choice option. |
| Tsai et al 2022 (60) | Survey results indicated conversational efficiency was improved by having a streamlined query mode. |
| Nassar et al 2023 (54) | Participants expressed a desire for more interactive dialogue, “I’d like to see a little more interaction” |
| Pienkowska et al 2023 (55) | Some users stated that they had negative perceptions of the chatbot, attributing this attitude to an inability to input free-text, making a participant "consider [them]self a passive participant" in the conversation. |

**Table 3:** Design features in the domain of Communication Style and sub-domain of Pacing which impacted user engagement

| **Author Year** | **Feedback** |
| --- | --- |
| Roca et al 2021 (56) | Some patients showed that they were keen to answer the virtual assistant, which may increase the attention and the responsibility of patients in taking their medication. Other patients who usually forgot to take their medication finally remembered to take it thanks to the alert on their mobile phones. |
| Balsa et al 2020 (47) | 10/242 comments pertained to amount of information as an aspect for further improvement of Vitória's design, such as ‘4th day: interaction with too much information’. |
| Balsa et al 2020 (47) | 16/242 comments pertained to repetition as an aspect for further improvement of Vitória's design, such as ‘daily questions about medication intake feel repetitive’. |
| Echeazarra et al 2021 (48) | Reminders and advice regularly sent to patients by the chatbot, along with the opportunity to review a video about best blood pressure measurement practices, appear to have had a positive impact on the knowledge and skills acquired in the intervention group. |
| Epalte et al 2020 (38) | One participant complained of “too much reading”. |
| Gingele et al 2022 (49) | 40% (n=10/25) of patients stated a preference for communicating with Molly once a week, 28% (n=7/25) of patients stated a preference for several times a week, and 8% (n=2/25) preferred daily contact. |
| ter Stal et al 2021 (58) | The frequency of messages, specifically, receiving too many messages from Sylvia led one participant to close the dialogue before reading. |
| Tongpeth et al 2018 (59) | Feedback from consumers indicated the length of time to complete a session was suitable. |
| Nassar et al 2023 (54) | Intervention pacing was approved, "the content and length are just right". |

**Table 4:** Design features in the domain of Communication Style and sub-domain of Redundancy which impacted user engagement

| **Author Year** | **Feedback** |
| --- | --- |
| Roca et al 2021 (56) | A patient was not able to answer medication reminders confirming they had taken their medicine, this was because the agent did not understand their messages, the authors observed 34 interactions that were not understood between the specific patient and the agent. However, the patient was able to indicate manually that they had taken medications. |
| Balsa et al 2020 (47) | 16/242 comments pertained to clarification as an aspect for further improvement of Vitória's design, such as “clarify what ‘two doses of alcohol’ means”. |
| Roca et al 2021 (56) | One patient had problems incorrectly answering reminders from the agent. However, when a second reminder was sent ten minutes later, the patient was able to answer correctly. |
| Roca et al 2021 (56) | The agent had the ability to customize medication regimens, which certain patients failed to update after initial configuration. Authors speculated several reasons for this, “forgetting to do so, being unaware of needing to do so, etc”. As a consequence, the agent stopped sending reminders about certain medications. |
| Guhl et al 2020 (51) | One participant liked the altered delivery of information from Tanya, “You can bring up information on Afib on the internet and read most of the same stuff on your own, but with Tanya you have a structured presentation that you're led through, so you end up getting the information you should be getting”. |
| Sagstad et al 2022 (57) | The information which users sought tended to be on information already provided by the healthcare service, indicating they preferred using the chatbot to access this sort of information. |
| Nassar et al 2023 (54) | Participants expressed a desire for more varied questions, “Chats are a bit repetitive” |
| Nassar et al 2023 (54) | Users approved of the inclusion of multi-media communication, "The visuals and videos help a lot". |

**Table 5:** Design features in the domain of Functionality and sub-domain of Miscellaneous which impacted user engagement

| **Author Year** | **Feedback** |
| --- | --- |
| Gong et al 2020 (50) | Some gamification elements and human-like characteristics were utilized in the My Diabetes Coach program design, as suggested by previous studies. The recently published and separate evaluation from My Diabetes Coach users demonstrated that these techniques increased users’ engagement with the program. |
| Tongpeth et al 2018 (59) | Consumers indicated the warning sign quiz and heart attack action quiz were helpful for understanding the heart attack action plan. |
| Roca et al 2021 (56) | 13 out of 13 patients gave an affirmative response to the statement “From a certain moment, the virtual assistant began to give the weather forecast. Has this helped you to use the virtual assistant more frequently?” |
| Nassar et al 2023 (54) | Participants approved of the interventions ability to quickly identify clinical and safety issues via dashboard flags which alerted the team to a need for timely intervention, “I like that it connects me to my doctor when things are not really right” |

**Table 6:** Design features in the domain of Functionality and sub-domain of Embodiment which impacted user engagement

| **Author Year** | **Feedback** |
| --- | --- |
| Apergi et al 2021 (37) | The embodiment of the conversational agent for the Avatar patients did not significantly change how often the patients used the technology compared to the Alexa+ patients, which had no visual appearance (p=0.76). |
| Baptista et al 2020 (36) | Engaging with Laura as an embodied conversational agent was reported to add a new layer to the communicative relationship, “instead of reading it, you're hearing it”. |

**Table 7:** Design features in the domain of Functionality and sub-domain of Personalization which impacted user engagement

| **Author Year** | **Feedback** |
| --- | --- |
| Guhl et al 2020 (51) | Authors speculated that the limited scope of content ‘focused on education, anticoagulant adherence, and symptom identification’, reduced engagement with their app, the content which was available was described as reasonably repetitive, median score of 5 on a Likert scale with 7 being “very repetitious”. |
| Guhl et al 2020 (51) | More participants completed the AF education module (79%) than the medications adherence counseling module (70%). |
| ter Stal et al 2021 (58) | Two participants indicated Sylvia communicated unwanted information. A participant stated that Sylvia would begin to talk about a topic, whether or not they were interested. Another participant only wanted advice when they were feeling unwell. Another participant expressed frustration, “When I have to go to work, I do not have time to watch a 15-minute video”. Another participant expressed frustration, “Look, what really bothered me was that, in the morning, I turned on the device and [the agent] started with saying: 'Did you follow the instruction?' Well I did not see any instruction yet”. |

**Table 8:** Design features in the domain of Accessibility and sub-domain of Platforms which impacted user engagement

| **Author Year** | **Feedback** |
| --- | --- |
| Cheng et al 2018 (35) | 2/10 of elderly participants did not find Google Home's virtual assistant easy to use (8/10 did), and only 5/10 of participants agreed with the statement that they would use it frequently (3/10 were neutral). This could be attributed to it not being physically convenient (3/10), understandable in terms of language (2/10), or because technical support would be needed (5/10, with 1/10 neutral). |
| Cheng et al 2018 (35) | 8/10 of elderly participants answered yes to “do you prefer using Healthy Coping on the Google Home over a smartphone?” (1/10 answered no, 1/10 no preference). |
| Cheng et al 2018 (35) | Speaker functionality was credited by most participants with a favourable view of the app as the reason for its high usability. |
| Echeazarra et al 2021 (48) | The main reason given from the 1/6 of patients who stopped using TensioBot was that they had difficulties using a mobile phone. |
| Epalte et al 2020 (38) | One participant referred to the tripod necessary to hold the tablet up as a hindrance due to there being “no space to put it” in medical institutions and limited spaces to put it in the “home”. Additionally, another participant wished the program “was installed on [their] computer”. |
| Tsai et al 2022 (60) | Survey results indicated approval for usage of the chatbot not requiring installation of another app as it was contained in the LINE APP. |

**Table 9:** Design features in the domain of Accessibility and sub-domain of Tutorials which impacted user engagement

| **Author Year** | **Feedback** |
| --- | --- |
| Balsa et al 2019 (46) | Authors noted the need for training older people with type 2 diabetes in using conversational agent-enabled interventions, citing the quote “(People) are not used to use technology”. |
| Cheng et al 2018 (35) | A difficult time learning the commands was credited by most participants with a negative view of the app as a reason for their view. |
| Epalte et al 2020 (38) | Almost all participants encountered technical difficulties and more often needed help from a relative or at some times the contacts for technical support provided. Most participants had difficulties identifying the cause of the problem due to their lack of confidence in using technologies. |
| Gingele et al 2022 (49) | Despite detailed information provided at the out-patient department, older HF patients with little digital experience were often unable to use the avatar autonomously at home. |
| Roca et al 2021 (56) | The authors stated that patients required a certain level of digital literacy, or to have assistance from people with this level, in order to configure the messaging platform. For example, being unable to turn on the sound of reminders after accidentally disabling them. |

**Table 10:** Design features in the domain of Visual Appearance and sub-domain of Anthropomorphism which impacted user engagement

| **Author Year** | **Feedback** |
| --- | --- |
| Balsa et al 2019 (46) | A negative aspect reported under the technology theme; ‘Facial expressions are weird’. |
| Baptista et al 2020 (36) | Laura’s “artificial movements” may have been the reason survey respondents described Laura as annoying (30%) and not real (27%). An additional complaint pertained to the dissonance between what was being said and her body movements. |
| Baptista et al 2020 (36) | Laura's human character was preferred to a cartoon character by some participants due to the nature of the conversations “I’m not sure I would have given the same level of credibility to, for example, a dog or a cat or something like that”. Conversely, those who expressed a desire for a cartoon character were motivated by it being more “fun”, which seemed to be more closely linked with perceived ease of use. |
| ter Stal et al 2021 (58) | One participant preferred to interact with a photo-realistic nurse, instead of a computer-animated figure, because a photo-realistic nurse would make the interaction more personal. The participant also stated: “I am not impressed by a cartoon figure”. Furthermore, the participant described the agent as “a male or female such as on the doors of bathrooms”. Another participant also preferred the agent to look like a nurse; this participant particularly commented on the agent’s clothing, “Put a white coat and a stethoscope on her”. |
| Tongpeth et al 2018 (59) | Feedback from consumers indicated they would have liked more natural gestures from Cora. |
| Pienkowska et al 2023 (55) | 3/8 participants had a strong positive stance regarding the appearance of the chatbot, with one participant who had a negative stance regarding it as "kitsch". |

**Table 11:** Design features in the domain of Visual Appearance and sub-domain of Gender which impacted user engagement

| **Author Year** | **Feedback** |
| --- | --- |
| ter Stal et al 2021 (58) | Many users made negative comments about Sylvia which referenced her gender. For example, “I found this female extremely annoying”, “Sylvia was a very irritating woman”, “a stupid woman”, and “She could be your girl next door...If I have medical complaints, I prefer an authority to explain what to do or not to do”. |
| ter Stal et al 2021 (58) | One participant liked that Sylvia was a woman as they “hated” listening to men. |

**Table 12:** Design features in the domain of Personality and sub-domain of Anthropomorphism which impacted user engagement

| **Author Year** | **Feedback** |
| --- | --- |
| Baptista et al 2020 (36) | Human-like characteristics were utilized in the My Diabetes Coach program design, as suggested by previous studies. A recently published and separate evaluation of users demonstrated that the technique increased users’ engagement with the program. |
| Baptista et al 2020 (36) | An agent backstory for Laura led to frustration for some users. |

**Table 13:** Design features in the domain of Personality and sub-domain of Mentorship which impacted user engagement

| **Author Year** | **Feedback** |
| --- | --- |
| Baptista et al 2020 (36) | Participants who saw Laura as an authority figure reported feeling guilty (17% of survey respondents). This was evidenced by one participant's account of feeling guilty, which caused him to cease using the app temporarily. Another participant considered presenting only her best readings to Laura to avoid “getting told off”. |
| Baptista et al 2020 (36) | Participants who viewed Laura as a friendly (61% of survey respondents) coach (supportive and non-blaming language) tended to report that interactions with Laura made them feel comfortable (36% of survey respondents) and engaged. This was evidenced by the tendency of survey respondents who described Laura as likable (61% of survey respondents) also reporting that interactions with Laura made them feel comfortable. |
| Baptista et al 2020 (36) | Regular emotional support, lacking in usual care, provided by Laura enhanced perceived usefulness. Evidenced by quotes expressing this need “I needed somebody just to be there”. Additionally, Laura's ability to monitor and provide positive reinforcement contributed to positive comments regarding her usefulness, “She was keeping you on track” and “I wasn't trying to be impressive for (Laura), but I think it just gave that little bit more incentive”. |
| Guhl et al 2020 (51) | One participant stated that she liked the fact that Tanya did not say “very drastic things” or make her feel “afraid”. Another referred to interacting with Tanya as “do[ing] something simple daily”. |
| ter Stal et al 2021 (58) | One participant expressed a preference for an authority figure over a more relatable figure in healthcare contexts, “She could be your girl next door...If I have medical complaints, I prefer an authority to explain what to do or not to do”. |
| Tongpeth et al 2018 (59) | Feedback from consumers indicated Nurse Cora was kind, friendly, and fun. Nurse Cora also received high ratings for ease of use. |
| Pienkowska et al 2023 (55) | Most users stated that the chatbot was more engaging than other educational modules within the app, with some attributing this to the chatbot feeling friendly and providing more personalized care. |

**Table 14:** Proposed design features in the domain of Accessibility

| **Author Year** | **Feedback** |
| --- | --- |
| Balsa et al 2020 (47) | 20/242 comments pertained to technology as an aspect for further improvement of Vitória's design, such as “buttons for recording medication are too small”. |
| Tongpeth et al 2018 (59) | Consumers indicated that the touch screen and navigation were easy to use. However, feedback from consumers indicated they would have liked instructions on how to navigate the app containing Cora at the beginning. |
| Pienkowska et al 2023 (55) | A participant suggested that should a notification advise a user to complete a certain module, an estimated completion time should be provided so the person knows if it is appropriate to complete in their current circumstance, "maybe you can include a time given for the module". This could also be relevant to content delivered via chatbot, for example, beginning a chat with 'I would like to ask you some questions, this should take 3 minutes'. |

**Table 15:** Proposed design features in the domain of Communication Style

| **Author Year** | **Feedback** |
| --- | --- |
| Balsa et al 2020 (47) | 14/242 comments pertained to physical activity as an aspect for further improvement of Vitória's design, such as ‘include “tiredness” as a possible reason for not having met the daily steps goal’. |
| Balsa et al 2020 (47) | 7/242 comments pertained to meals as an aspect for further improvement of Vitória's design, such as ‘(. . . ) it doesn’t include soup. Most elders eat soup at meals’. |
| Epalte et al 2020 (38) | The multiple-choice options to respond led to various issues for participants, one stating “there are some that might interest equally”, referring to losing the ability to choose an option if another is chosen. Additionally, another found it “strange” when “there is only one option” and “nothing to choose”. One participant even advocated for being able to “write directly” to allow them to “ask a question”, another complained that they lacked the “opportunity to ask questions”. |
| Gingele et al 2022 (49) | 3/25 of patients suggested Molly should leave out punctuation marks when speaking. |
| Gingele et al 2022 (49) | 5/25 of patients suggested Molly should have voice recognition. |
| Tongpeth et al 2018 (59) | Feedback from consumers indicated they would have liked the ability to repeat dialogue. |
| Tongpeth et al 2018 (59) | Feedback from consumers indicated they would have liked larger font size to emphasize important messages while Nurse Cora talked. |
| Nassar et al 2023 (54) | Participants expressed a desire for more information about specific subjects, “Add a feature that answers specific questions |
| Pienkowska et al 2023 (55) | Participants proposed that the chatbot should have an option to rerun a conversation |
| Pienkowska et al 2023 (55) | Participants proposed that the chatbot should have a free-text option, "I mean, you guys didn't factor in, like, you know, that people ask questions". |

**Table 16:** Proposed design features in the domain of Functionality

| **Author Year** | **Feedback** |
| --- | --- |
| Balsa et al 2020 (47) | 21/242 comments pertained to miscellaneous areas as an aspect for further improvement of Vitória's design, such as ‘reply options for the user always affirmative, without option for refusal’ or the fact that it is not possible to repeat the interaction more than once every day. |
| Balsa et al 2020 (47) | 53/242 comments pertained to new functionalities as an aspect for further improvement of Vitória's design, such as developing a ‘strategy to remind users to use the app’ and advocating for the ‘development of recipes for people with diabetes”. |
| ter Stal et al 2021 (58) | Participants suggested Sylvia should confirm details or follow up on certain topics, one participant suggesting “But, then, ask the next day: 'Did you read that? Did you do this?'“. |
| Tongpeth et al 2018 (59) | Feedback from consumers indicated they would have liked more feedback during the quizzes. |
| Pienkowska et al 2023 (55) | Participants proposed that the chatbot should have an option to search topics. |
| Pienkowska et al 2023 (55) | Participants proposed that the chatbot should be integrated with a human, in that conversations can be escalated to a human if the chatbot is unable to assist with something. |

**Table 17:** Research gaps in the domain of Unstated features

| **Author Year** | **Feedback** |
| --- | --- |
| Balsa et al 2019 (46) | A minimum score of 4 on the Likert scale (5 = strongly agree) among type 2 diabetes patients agreeing with the statement that the application was easy to use. The reason for this was not explained, though agreement with the statement that characters on the screen could be read well was graded at a minimum of 4 by type 2 diabetes patients. |
| Balsa et al 2019 (46) | Minimum score of 4 on the Likert scale (5 = strongly agree) among type 2 diabetes patients agreeing with the statement that “I would like to try the application for a few weeks”. |
| Balsa et al 2019 (46) | Patients were satisfied with Vitória’s final feedback on their answers to questions posed in the evaluation phase (mean 4.56, min 4 max 5). |
| Balsa et al 2019 (46) | A user comment under the category intuitiveness: “Easy to handle and understand”. The statement ‘I would like to try the application for a few weeks’ received positive answers from patients (mean 4,56, min 4 max 5). |
| Echeazarra et al 2021 (48) | When asked about ease of use, participants responded with very easy (n=23), pretty easy (n=14), or quite difficult (n=3). Additionally, 92.5% (n=37/40) preferred to register their blood pressure with TensioBot instead of paper. |
| Echeazarra et al 2021 (48) | When asked about usefulness, participants responded with very useful (n=29), or quite useful (n=11). Additionally, 85% (n=47/55) of patients who successfully participated in the intervention arm of the experiment fully, continued to use TensioBot after the experiment ended. |
| Epalte et al 2020 (38) | Changes to the user interface made in the second iteration of Dina, including providing more options to click on related themes to specific conversation, ‘guiding’ the user through the conversation, may have contributed to changes in certain data analytics. Analytics indicated more efficient communications, long dialogues (4-6 questions) were significantly less frequent during the second iteration, 17.7% (187/322) vs 53.1% (153/288), p=0.013 |
| Epalte et al 2020 (38) | Changes to the user interface made in the second iteration of Dina, may have contributed to changes in certain data analytics. Analytics indicated more clear communications, questions on manoeuvring and orienting in the chatbot were significantly less frequent during the second iteration, 6.69% (101/1509) vs 18.28% (243/1329), p<0.001. |
| Epalte et al 2020 (38) | Changes to the user interface made in the second iteration of Dina, may have contributed to changes in certain data analytics. Analytics indicated more satisfactory communications, fallback questions which were not understood were significantly less frequent during the second iteration, 18.2% (31/17) vs 32.1% (50/156), p=0.004. |
| Epalte et al 2020 (38) | One participant stated “Chat is also very useful”. |
| Epalte et al 2020 (38) | Patients were pleased with the home-based program and stated that it motivated them to be more active and participate in activities that benefit their health. |
| Gong et al 2020 (50) | Of the 66 out of 93 participants who responded to the 6-month process evaluation, over 80% considered Laura as a helpful, friendly and competent assistant. |
| Gong et al 2020 (50) | The decreasing trend in program use over time and the dose-response relationship between the level of app use and its effectiveness suggest that more efforts are still required to improve the maintenance of program use over time. |
| Guhl et al 2020 (51) | Participants indicated talking with the agent was easy, median score of 1 on a Likert scale, 1 being easy, 7 being difficult. An explanation from the users for this rating, in terms of design features was not provided. Additionally, one participant stated that “talking to Tanya” could help them “do something for myself”. |
| Kimani et al 2016 (52) | A total of 11 participants completed a self-report rating of the agent. Responding to the question, “How easy was talking to the agent?” they provided a rating from 1 to 4, where 1 signified “very difficult” and 4 meant “very easy.” The average mean score from these responses was 3.54 (standard deviation = 0.69). |
| Kimani et al 2016 (52) | A total of 11 participants completed a self-report rating of the agent. Responding to the question, “How helpful was the agent to you?” they provided a rating from 1 to 4, where 1 signified “not at all helpful” and 4 meant “very helpful.” The average mean score from these responses was 2.82 (standard deviation = 0.98). |
| Kimani et al 2016 (52) | Participants in the study found the atrial fibrillation information to be helpful and were relatively satisfied with their interaction with the agent. |
| Magnani et al 2017 (53) | Participants responding to the statement, “I am confident in Tanya’s ability to help me.” with a rating on a scale from 1 to 7, where 1 signified “disagree completely” and 7 meant “agree completely”. The median average score from these responses was 5, ranging from 3-7. |
| Magnani et al 2017 (53) | Participants responding to the question, “How easy was talking to Tanya?” with a rating on a scale from 1 to 7, where 1 signified “easy” and 7 meant “difficult”. The median average score from these responses was 1, ranging from 1-7. |
| Magnani et al 2017 (53) | The responses to a postsurvey acceptability assessment suggested that users found the agent accessible, informative, and trustworthy. |
| Roca et al 2021 (56) | 12 out of 13 patients gave an affirmative response to the statement “Did you find it easy to use the virtual assistant?” |
| Roca et al 2021 (56) | 12 out of 13 patients gave an affirmative response to the statement “Do you think [the agent] is useful for you?”. Additionally, 12 out of 13 patients gave an affirmative response to the statement “Do you think the virtual assistant improves your medication adherence?” |
| Roca et al 2021 (56) | The experience with the virtual assistant seems to have been very positive, as observed in the patients’ opinions and interactions throughout the study. Almost 70% of the patients (9/13) agreed with the idea of continuing using the virtual assistant after the study. |
| ter Stal et al 2021 (58) | At the end of the 9-week intervention participants (n=8) gave Sylvia a median rating of 5.0 (IQR: 4.0-7.0) in terms of expertise. At the end of the 9-week intervention participants (n=9) gave Sylvia a median rating of 4.0 (IQR: 3.8-7.0) in terms of reliability. At the end of the 9-week intervention participants (n=9) gave Sylvia a median rating of 4.0 (IQR: 2.5-5.0) in terms of 'Likeliness of following advice'. |
| ter Stal et al 2021 (58) | In the interviews, 15 weeks from baseline, the majority of the participants (n=7) did not think Sylvia had value, “Sylvia is not it”. |
| Tongpeth et al 2018 (59) | The ten consumers agreed (80%), or strongly agreed (20%), that the app was easy to use. |
| Zhang et al 2015 (61) | Patients were highly satisfied with the virtual counselor, exhibited high desire to continue working with her, trusted and liked her, and would follow her advice in the future. |
| Zhang et al 2015 (61) | When asked to respond to the question “How easy was talking to the counselor/doctor?” on a scale of 'easy' (1) to 'difficult' (7), participants rated the agent 2.17 (standard dev. = 1.94). For comparison, the human doctor received a rating of 1.25 (standard dev. = 0.50). |
| Zhang et al 2015 (61) | When asked to respond to the question “How much would like to continue working with the counselor/doctor?” on a scale of 'not at all' (1) to 'very much' (7), participants rated the agent 4.83 (standard dev. = 1.60). For comparison, the human doctor received a rating of 6.50 (standard dev. = 0.58). |
| Nassar et al 2023 (54) | 47 out of 48 participants responded to a survey that they found the diabetes chats easy to use. However, this finding could not be directly cross referenced to specific features elaborated in postchat comments. |
| Nassar et al 2023 (54) | Unstated feature resulted in a positive change to perceived effectiveness: 42 out of 48 participants responded to a survey that they felt diabetes chats made more confident in managing their health. However, this finding could not be directly cross referenced to specific features elaborated in postchat comments. |
| Pienkowska et al 2023 (55) | To varying degrees, all participants were willing to recommend the chatbot to others, though the reason for this was not directly interrogated. |

**Table 18:** Research gaps in the domain of Unstated features

| **Author Year** | **Feedback** |
| --- | --- |
| Apergi et al 2021 (37) | Linear regression models of study data indicated black patients used the conversational agent-enabled interventions an average of 21 times less than non-Black patients who had similar characteristics otherwise (p=0.02). |
| Apergi et al 2021 (37) | Linear regression models of study data indicated patients managing heart failure who were on a larger number of medications tended to engage with the conversational agent-enabled interventions less frequently on average (p=0.002). |
| Apergi et al 2021 (37) | Linear regression models of study data indicated patients managing heart failure who were older tended to engage with the conversational agent-enabled interventions more frequently on average (p=0.004). |
| Zhang et al 2015 (61) | Participants who were high school graduates or higher had significantly more trust in the agent than those without high school degrees (p<0.05). |
| Zhang et al 2015 (61) | Participants with high computer literacy felt a greater sense of care from the agent than those with low computer literacy (p<0.05). |
